# FQ-UWF: Unpaired Generative Image Enhancement for Fundus Quality Ultra-Widefield Retinal Images

**DOI:** 10.3390/bioengineering11060568

**Published:** 2024-06-04

**Authors:** Kang Geon Lee, Su Jeong Song, Soochahn Lee, Bo Hee Kim, Mingui Kong, Kyoung Mu Lee

**Affiliations:** 1 Department of Electrical and Computer Engineering, Automation and Systems Research Institute (ASRI), Seoul National University, Seoul 08826, Republic of Korea; dlrkdrjs97@snu.ac.kr; 2 Department of Ophthalmology, Kangbuk Samsung Hospital, Sungkyunkwan University School of Medicine, Seoul 03181, Republic of Korea; sjsong7@gmail.com (S.J.S.); puhaha13@gmail.com (B.H.K.); eyedockong@gmail.com (M.K.); 3 Biomedical Institute for Convergence (BICS), Sungkyunkwan University, Suwon 16419, Republic of Korea; 4 School of Electrical Engineering, Kookmin University, Seoul 02707, Republic of Korea; sclee@kookmin.ac.kr; 5 Interdisciplinary Program in Artificial Intelligence, Seoul National University, Seoul 08826, Republic of Korea

**Keywords:** unpaired super-resolution, retinal fundus image enhancement, ultra-widefield retinal image

## Abstract

Ultra-widefield (UWF) retinal imaging stands as a pivotal modality for detecting major eye diseases such as diabetic retinopathy and retinal detachment. However, UWF exhibits a well-documented limitation in terms of low resolution and artifacts in the macular area, thereby constraining its clinical diagnostic accuracy, particularly for macular diseases like age-related macular degeneration. Conventional supervised super-resolution techniques aim to address this limitation by enhancing the resolution of the macular region through the utilization of meticulously paired and aligned fundus image ground truths. However, obtaining such refined paired ground truths is a formidable challenge. To tackle this issue, we propose an unpaired, degradation-aware, super-resolution technique for enhancing UWF retinal images. Our approach leverages recent advancements in deep learning: specifically, by employing generative adversarial networks and attention mechanisms. Notably, our method excels at enhancing and super-resolving UWF images without relying on paired, clean ground truths. Through extensive experimentation and evaluation, we demonstrate that our approach not only produces visually pleasing results but also establishes state-of-the-art performance in enhancing and super-resolving UWF retinal images. We anticipate that our method will contribute to improving the accuracy of clinical assessments and treatments, ultimately leading to better patient outcomes.

## 1. Introduction

**Figure 1:**
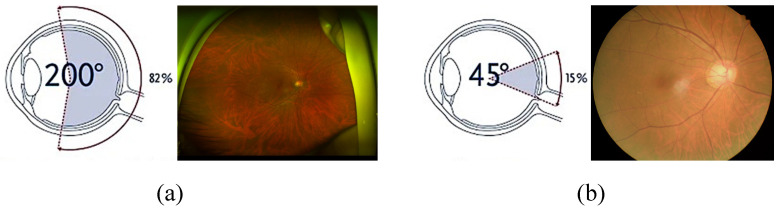
Conventional fundus image vs. ultra-widefield (UWF) image. (**a**) UWF images drastically increase the capability to observe the retina and can cover over 80%, which is more than a five-fold increase compared to (**b**) conventional fundus images. The diagrams in the left of (**a**,**b**) are reproduced from https://www.optomap.com/optomap-imaging/ accessed on 1 March 2022.

Ultra-widefield (UWF) retinal images have emerged as a revolutionary modality in ophthalmology [1,2]. As depicted in Figure 1, UWF provides an extensive field of view that enables the visualization of both central and peripheral retinal areas. This enables early detection and monitoring of peripheral retinal conditions that are often missed in standard fundus images. However, various artifacts, low macular area resolution, large data size, and lack of interpretation standardization act as impediments to widespread clinical use of UWF images.

Image enhancement techniques have the potential to improve UWF image quality, empowering healthcare professionals to make more accurate diagnoses and treatment plans. Ophthalmologists may better detect subtle early changes in the macular area and identify peripheral early signs of disease, leading to better patient outcomes. But since UWF images contain multiple degradation factors scattered throughout the fundus in a complex manner, image enhancement is a significant challenge. Many recent conventional image enhancement techniques are based on supervised learning and require a ground truth (GT) dataset of well-aligned low- and high-quality image pairs for training. Achieving this paired dataset is a significant challenge in the case of UWF, where precise alignment between image pairs is extremely difficult.

The application of deep learning algorithms has facilitated promising results in a wide range of image enhancement tasks, including super-resolution, image denoising, and image deblurring [3]. A variety of methods tailored for enhancement of retinal fundus images have also been proposed [4,5]. These methods can automatically learn and apply complex transformations to improve the visualization of critical structures such as blood vessels, the optic disc, and the macula. Despite the necessity, there has yet to be a comprehensive deep-learning-based enhancement method for UWF images.

**Figure 2:**
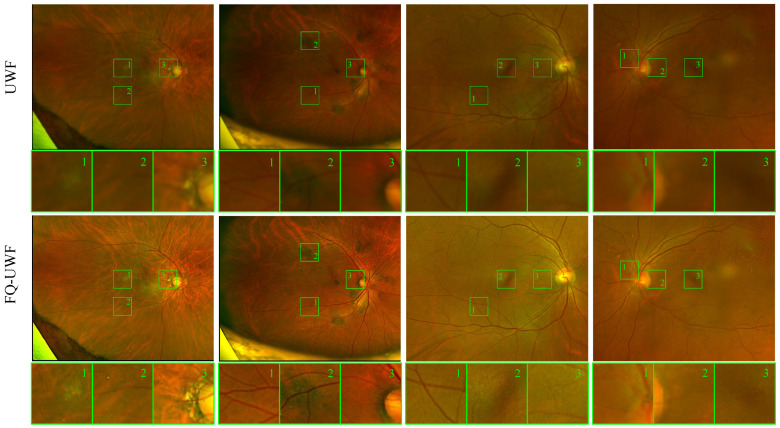
Sample results of the proposed UWF enhancement method. The top row depicts the input UWF images, and the bottom row depicts the FQ-UWF images enhanced by the proposed method. Numbered boxes are enlarged sample views of representative local regions. The clarity of anatomical structures such as vessels is greatly improved in the FQ-UWF images.

We thus propose a comprehensive image enhancement method for UWF images, with the specific goal of improving the quality of conventional fundus images. Figure 2 presents sample results of the proposed method. As image quality can be subjective, we compare manual annotations of drusen from fundus images and UWF images after applying our enhancement method. Experimental evaluation demonstrates that the similarity between annotations after enhancement is considerably improved compared to annotations made on images before enhancement. Quantitative measurements of image quality are also assessed, demonstrating state-of-the-art results on several datasets. Based on our goal and the experimental findings, we refer to the enhanced images as fundus quality (FQ)-UWF images. We believe that our approach has the potential to improve the accuracy of clinical assessments and treatments, ultimately leading to better patient outcomes.

The proposed method is based on the generative adversarial network (GAN) framework to avoid the requirement of pairs of aligned high-quality images in pixelwise supervision. We employ a dual-GAN structure to jointly perform super-resolution, enhancing the low resolution of the macula in UWF, which has a critical impact on clinical practice. As image pairs are not required, training data are acquired by simply collecting sets of UWF and fundus images. We also incorporate appropriate attention mechanisms in the network for enhancement with regard to various degradations such as noise, blurring, and artifacts scattered throughout the UWF.

We summarize our contributions as follows:

- We establish a method for UWF image enhancement and super-resolution from unpaired UWF and fundus image sets. We evaluate the clinical utility in the context of detecting and localizing drusen in the macula.
- We propose a novel dual-GAN network architecture capable of effectively addressing diverse degradations in the retina while simultaneously enhancing the resolution of UWF images.
- The proposed method is designed to be trained on unpaired sets of UWF and fundus images. We further present a corresponding multi-step training scheme that combines transfer learning and end-to-end dataset adaptation, leading to enhanced performance in both quantitative and qualitative evaluations.

## 2. Related Works

### 2.1. Retinal Image Enhancement

Due to the relatively invariable appearance, methods based on traditional image processing techniques continue to be proposed [6,7]. But the majority of methods leverage deep neural networks, as in [5,8], and especially GANs in particular [4].

Pham and Shin [9] considered additional factors such as drusen segmentation masks to not only improve image quality but also preserve crucial disease information during the enhancement process, addressing a common challenge in existing image enhancement techniques. To overcome the challenges of constructing a clean true ground truth (GT) dataset for retinal image data, particularly due to factors such as alignment, Yang et al. [4] introduced an unpaired image generation method for enhancing low-quality retinal fundus images. Lee et al. [5] proposed an attention module designed to automatically enhance low-quality retinal fundus images afflicted by complex degradation based on the specific nature of their degradation.

### 2.2. Blind and Unpaired Image Restoration

Blind image restoration is a computational process aimed at enhancing or recovering degraded images without prior knowledge of the degradation model or parameters. Traditionally, methods for blind image restoration have employed approaches involving the prediction of the estimation of degradation model parameters [10] or the degradation kernels [11]. Recently, there has been a trend towards directly generating high-quality images through training using deep learning models [12]. Shocher et al. [13] conducted super-resolution without relying on specific training examples of the target resolution during the model’s training phase. Yu et al. [14] proposed a blind image restoration toolchain for multiple tasks with reinforcement learning.

Unpaired image restoration focuses on learning the difference between pairs of image domains rather than pairs of individual images. Multiple methods using GAN-based models [15] have been proposed [16,17] to learn the mapping between the low-quality and high-quality images while also incorporating a cycle-consistency constraint [18] to improve the quality of the generated images.

### 2.3. Hierarchical or Multi-Structured GAN

Recently, there has been significant progress in mitigating the instability associated with GAN training, leading to the emergence of various proposed approaches that involve connecting two or more GANs for joint learning. Several works showed stable translation between two different image domains using coupled-GAN architectures [19]. Further works extended their usage to multiple domains or modalities [20,21]. And more works extended this approach beyond random image generation to tasks such as image restoration [16], and exploration into more complex architectures has also been proposed [22].

### 2.4. Transfer Learning for GANs

Pre-trained GAN models have demonstrated considerable efficacy across various computer vision tasks, particularly in scenarios characterized by limited training data [23,24]. Typically trained on extensive datasets comprising millions of annotated images, these models offer a foundation of learned features. Through the process of fine-tuning on novel datasets, one can capitalize on these pre-trained features, leading to the attainment of state-of-the-art performance across a diverse spectrum of tasks.

Early works confirmed successful generation in a new domain by transferring a pre-trained GAN to a new dataset [25,26]. Other works enabled transfer learning for GANs with datasets of limited size [27,28]. Li et al. [29] proposed an optimization method for transfer learning for GAN that was free from biases towards specific classes and resilient to mode collapse and achieved by fine-tuning only the class embedding layer, which is part of the GAN architecture. Mo et al. [30] proposed a method wherein the lower layers of the discriminator are fixed; then, it is partitioned into a feature extractor and a classifier. Subsequently, only the classifier is fine-tuned. Fregier and Gouray [26] performed transfer learning for GAN on a new dataset by freezing the low-level layers of the encoder, thereby preserving pre-trained knowledge to the maximum extent possible.

## 3. Methods

### 3.1. Overview of FQ-UWF Generation

**Figure 3:**
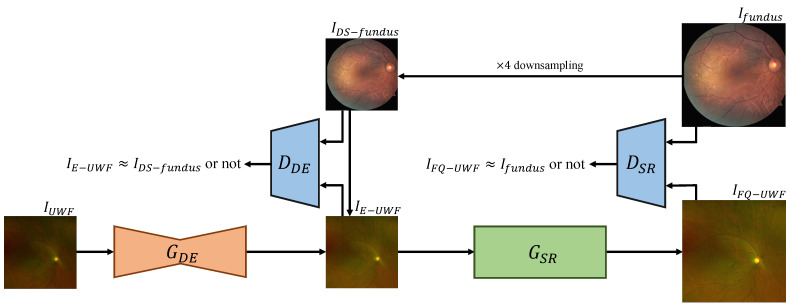
The overall architecture of the proposed method. $I_{UWF}$ with severe degradations and artifacts is first enhanced to $I_{E-UWF}$ via $G_{DE}$, for which the output is fed to $G_{SR}$ to generate ×4 up-scaled $I_{FQ-UWF}$. $I_{fundus}$ is down-scaled to $I_{DS-fundus}$ with a scaling factor of 4. $D_{DE}$ and $D_{SR}$ measure the similarity between $I_{E-UWF}$ and $I_{DS-fundus}$ to train $G_{DE}$ and the similarity between $I_{FQ-UWF}$ and $I_{fundus}$ to train $G_{SR}$, respectively.

To get a final enhanced FQ-UWF result $I_{FQ-UWF}$, we split the process of FQ-UWF generation into two steps: (i) degradation enhancement (DE) and (ii) super-resolution (SR). Figure 3 presents a visual overview of the framework. The order of the processes is tailored to maximize the quality of the output FQ-UWF images. The generator networks of each process, which we respectively denote as $G_{DE}$ and $G_{SR}$, are coupled with adversarial discriminator networks $D_{DE}$ and $D_{SR}$ that are designed to enforce that the generators’ output images have similar image characteristics as the fundus images from the training set.

$G_{DE}$ performs degradation enhancement on input image $I_{UWF}$ to get $I_{DE-UWF}$. Training of $G_{DE}$ is guided by $D_{DE}$ so that the $D_{DE}$ output score values are similar for the given pair of $I_{E-UWF}$ and $I_{DS-fundus}$, which is a ×4 bicubically downsampled version of $I_{fundus}$. $D_{DE}$ is trained to make the score value of the given pair of images significantly differ.

$G_{SR}$ performs ×4 super-resolution on $I_{E-UWF}$ to get $I_{FQ-UWF}$. $G_{SR}$ and $D_{SR}$ are trained in the same manner as $G_{DE}$ and $D_{DE}$, respectively, with the pair of $I_{FQ-UWF}$ and $I_{fundus}$. For $D_{SR}$, we also impose cyclic constraints, as in [18,31], by applying the $G_{SR}$ operation to not only $I_{E-UWF}$ but to $I_{DS-fundus}$ as well. For each module, we empirically determined appropriate network architectures. The following subsections describe further details of each module.

### 3.2. Architecture Details

#### 3.2.1. $G_{DE}$

**Figure 4:**
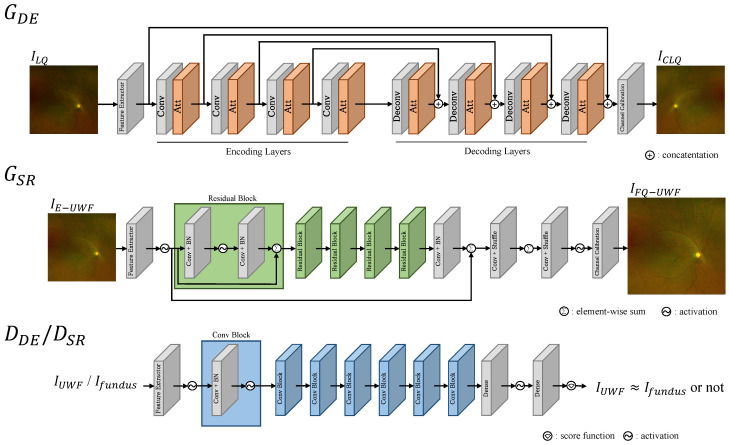
The detailed structure of generators and discriminators. The detailed structure of generator $G_{DE}$, $G_{SR}$, and the discriminator shared between $D_{DE}$ and $D_{SR}$ is illustrated. Note that even though $D_{DE}$ and $D_{SR}$ utilize the same structure, they are fundamentally distinct discriminative networks.

We apply U-net [32] as the base architecture, as U-net has been proven to be effective for medical image enhancement [33]. Within the encoder–decoder structure of U-net, we embed attention modules to better enhance local degradation or artifacts scattered throughout the input image. We apply the attention layer structure proposed by [5], as it has been demonstrated to be effective for retinal image enhancement. The network structure is depicted in the top row of Figure 4.

The Conv box comprises a 3×3 convolutional layer so that the spatial size of the feature is reduced to 1/4, where both the height and the width of the feature are reduced to 1/2, and the channel dimension is doubled. The Deconv box comprises a 3×3 deconvolutional layer so that the spatial size of the feature is quadrupled, where both the height and the width of the feature are doubled, and the channel dimension is halved. The attention (Att) box comprises a sequentially connected batch normalization, activation, operation-wise attention module, and activation, where the operation-wise attention module enables the degradations to be better attended.

#### 3.2.2. $G_{SR}$

The network structure is depicted in the middle row of Figure 4. The FeatureExtractor box comprises a 3×3 convolutional layer followed by activation. The Conv+BN box comprises a 3×3 convolutional layer followed by batch normalization. The Conv+Shuffle box comprises a 3×3 convolutional layer followed by a pixel shuffler for expanding the height and width of the feature by a factor of two each. Channel calibration is designed for reducing the dimension of the feature to three, maintaining the spatial dimension of the feature. The Residual Block comprises series of Conv+BN, activation, Conv+BN, and residual connections for element-wise summing. We note that this structure is adopted from [15].

#### 3.2.3. $D_{DE}\;and\;D_{SR}$

The structures of the discriminator models $D_{DE}$ and $D_{DE}$ are depicted in Figure 4. The FeatureExtractor box comprises a 3×3 convolutional layer followed by activation. The Conv+BN box comprises a 3×3 convolutional layer followed by batch normalization. The Conv Block comprises series of Conv+BN and activation. At the final layer of the network, there exists a score function for evaluating the similarity of input images, accompanied by a Dense layer aimed at reducing the dimension of the feature to a single scalar score value. We follow the structure of the discriminator in [15] for $D_{DE}$. The input images for $D_{DE}$ are pairs of downsampled real fundus images $I_{DS-fundus}$ and generated enhanced low-resolution UWF images $I_{E-UWF}$. The input images for $D_{SR}$ are pairs of real fundus images $I_{fundus}$ and generated FQ-UWF images $I_{FQ-UWF}$.

### 3.3. Loss Functions and Training Details

Given that end-to-end training of an architecture composed of multiple networks is highly challenging, we take three steps to train the full network architecture composed of (i) $G_{DE}$ training, (ii) $G_{SR}$ training, and (iii) overall fine-tuning.

#### 3.3.1. $G_{DE}$ Training

We first impose adversarial loss on $G_{DE}$ and $D_{DE}$ as follows:

(1) $$\begin{array}{cc} L_{L}\;=\; & E_{x∼I_{DS-fundus}}\left[\log D_{DE}\left(x\right)\right]\\ \; & +E_{z∼I_{UWF}}\left[1-\log D_{DE}\left(G_{DE}\left(z\right)\right)\right]. \end{array}$$

The identity mapping loss is important when performing tasks such as super-resolution or enhancement, as it helps to maintain the style (color, structure, etc.) of the source domain’s image while applying the target domain’s information [18]. Thus, we use the loss function defined as:

(2) $$L_{I}=E_{z∼I_{UWF}}∥\;G_{DE}\left(z\right)-z\;∥.$$

We especially impose L2 regularization [34] loss $L_{R}$ on the weight of $G_{DE}$ to retain knowledge by preventing the abrupt change of the weight as much as possible when we use pre-trained $G_{DE}$ with other datasets. Finally, the loss function $L_{E}$ to adapt the $G_{DE}$ to the fundus-UWF retinal image dataset is defined as:

(3) $$\begin{array}{cc} L_{E}\; & =\;L_{L}+\lambda_{I}\;L_{I}+\lambda_{R}\;L_{R}, \end{array}$$

where $\lambda_{I}$ and $\lambda_{R}$ control the relative importance of $L_{I}$ and $L_{R}$, respectively.

For more efficient adversarial training, we initialize the network parameters by pretraining using [5]. We then freeze the encoder parameters and only update the decoder parameters.

#### 3.3.2. $G_{SR}$ Training

In this step, we freeze all trainable parameters in $G_{DE}$ to generate $I_{E-UWF}$ from $I_{UWF}$. After the adaptation process for $G_{DE}$ is done, we apply adversarial loss to $G_{SR}$, which takes $I_{E-UWF}$ from $G_{DE}$ as an input and outputs the FQ-UWF result $I_{FQ-UWF}$, which is defined as:

(4) $$\begin{array}{cc} L_{H}\;=\; & E_{x∼I_{fundus}}\left[\log D_{SR}\left(x\right)\right]\\ \; & +\;E_{z∼I_{E-UWF}}\left[1-\log D_{SR}\left(G_{SR}\left(z\right)\right)\right]. \end{array}$$

We also impose a cycle constraint [18], which maintains consistency between the two domains, resulting in more realistic and coherent image translations on $I_{fundus}$ → $I_{DS-fundus}$ → $I_{FQ-UWF}$. This can be denoted as follows:

(5) $$L_{C}=E_{x∼I_{fundus}}∥\;G_{SR}\left(D_{SR}\left(x\right)\right)-x\;∥.$$

As mentioned in [17], by applying one-way cycle loss, the network can learn to handle various degradations by opening up the possibility of one-to-many generation mapping.

Overall, the loss function for $G_{SR}$ training is expressed as follows:

(6) $$\begin{array}{cc} L_{R}\; & =\;L_{H}+\lambda_{C}\;L_{C}, \end{array}$$

where $\lambda_{C}$ controls the relative importance of $L_{C}$.

#### 3.3.3. Overall Fine-Tuning

In the previous training steps, $G_{DE}$ and $G_{SR}$ are trained independently. But to ensure stability and integration between the two generators, a final calibration process is performed on the entire architecture. Additionally, to improve the network’s performance in clinical situations, where the diagnosis of lesions is mainly based on the macular region rather than the periphery of the fundus, we again employ the same loss combinations as follows, only using patches from the macular region to fine-tune the entire model:

(7) $$L_{M}=L_{E}+L_{R}.$$

## 4. Experiments

### 4.1. Datasets and Settings

We used 3744 UWF images and 3744 fundus images acquired from the Kangbuk Samsung Medical Center (KBSMC) Ophthalmology Department from 2017 to 2019. Although UWF and fundus images were acquired in pairs, we anonymized and shuffled the image sets and did not use information of paired images during training. To train the model proposed in this paper, we used 3370 UWF and 3370 fundus images (unpaired). We set the scaling factor for super-resolution to 4, which was close to the approximate average difference in resolution between the UWF and fundus images. To test the model, we used 374 UWF images that were not used during training.

### 4.2. Implementation Details

We use the AdamW [35] optimizer with learning rate =1e−3, $\beta_{1}=0.9$, $\beta_{2}=0.999$, and $\epsilon =10^{-8}$ to train $G_{DE}$ and $G_{SR}$, with weight decay every 100K iterations with a decay rate of 1e−2. We set the learning rate to be halved every 200K iterations and the batch size as 16, and we train the model for more than $5\times 10^{6}$ iterations using an NVIDIA RTX 2080Ti GPU. We feed two 128×128-sized $I_{UWF}$ and $I_{fundus}$ patches that are randomly extracted from the UWF and fundus retinal images, respectively. During training, we apply additional dataset augmentations using rotation and flipping for $I_{UWF}$ and $I_{fundus}$.

We set $\lambda_{I}$, $\lambda_{R}$, and $\lambda_{C}$, which adjust the degree of importance of $L_{I}$, $L_{R}$, and $L_{C}$ to be 0.5, 0.1, and 0.5, respectively.

### 4.3. Baselines for Comparison

We choose the following baselines to compare with the proposed method on the KBSMC dataset: (i) ZSSR [13], (ii) *cycle-in-cycle* GAN [36], (iii) KMSR [37], (iv) CinCGAN [16], and (v) RLrestore [14] + bicubic upsampling. We train these five baselines on the KBSMC dataset from scratch.

### 4.4. Evaluation Metrics

As we do not assume paired images for training, we avoid the use of reference-based metrics such as the PSNR [38] or SSIM [39] that require paired GTs. Instead, we measure the LPIPS [40] and the FID [41]. Both metrics indicate a closer distance between the two images when their values are smaller.

Additionally, given the nature of retinal images with various degradations, achieving sharp images is also an important consideration. To measure this, we measure γ [42,43]. A lower value of the γ metric implies a higher level of sharpness in the generated images, and therefore, the model is considered to deliver higher performance. We further substantiate the statistical validity of our comparisons by employing two-sided tests. We first utilize ANOVA [44] to ascertain whether there were significant differences in the means among groups. Subsequently, to identify specific groups where differences exist, we employ Bonferroni’s correction [45]. These analyses are conducted using *p*-values for confirmation.

Furthermore, we attempt to measure the clinical impact of our method by comparative evaluation of the visibility of drusen in the $I_{UWF}$ images before improvement, the $I_{FQ-UWF}$ images after improvement, and the $I_{fundus}$ images. In this process, medical practitioners annotated drusen masks in the order of $I_{UWF}\to I_{FQ-UWF}\to I_{fundus}$ to minimize potential biases that might arise.

### 4.5. Experiments on the KBSMC Dataset

Figure 2 depicts samples of the enhancement by the proposed method. Improved clarity of vessel lines and background patterns can be observed.

#### 4.5.1. Domain Distance Measurement Results

**Figure 5:**
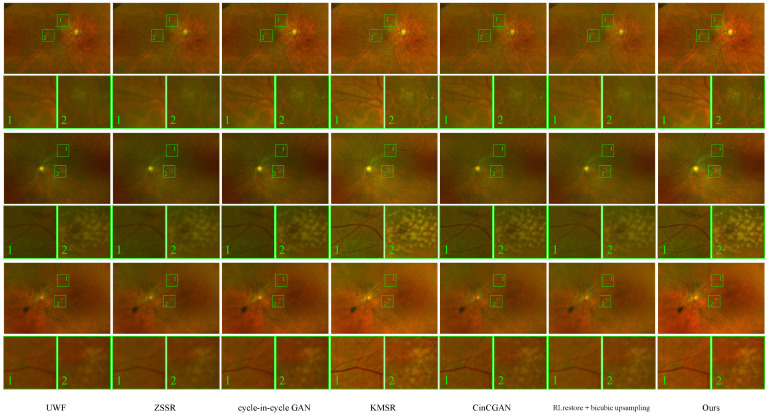
The enhanced FQ-UWF results. Input $I_{UWF}$ images are improved using various methods.

Table 1 shows the γ, LPIPS, and FID results of the baselines for comparison and our method. The proposed method yields the best results in terms of the γ and LPIPS metrics and the second-best results in terms of the FID. Figure 5 shows the corresponding sample results before and after the improvements with the given methods. We can see visible improvements in the patterns of vessels and the macula. This is corroborated by the γ values in Table 1. The low *p*-values <0.001 in the table show the statistical significance of our method in terms of LPIPS, FID, and γ.

#### 4.5.2. Enhancement Results for Severe Degradations

**Figure 6:**
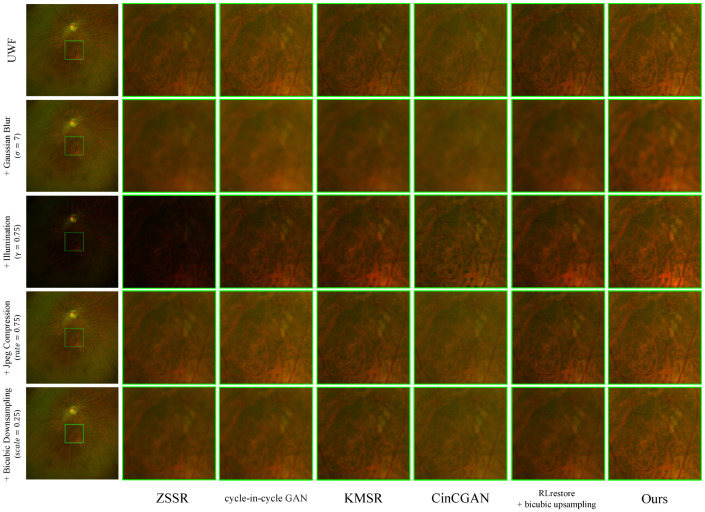
The enhanced FQ-UWF results. Different types of degradation are applied to $I_{UWF}$ images. Degraded images are improved using various methods.

Figure 6 illustrates the comparison with various unpaired super-resolution methods and our method for the challenging scenario wherein the input image is corrupted with the following synthetic degradations: (i) Gaussian blur with σ=7, where the image is degraded with a Gaussian blur kernel of size σ × σ as in [46]; (ii) Illumination with γ=0.75, where the brightness of the image is unevenly illuminated by gamma correction with γ as in [47]; (iii) JPEG compression with rate=0.25, where the compressionratio=rate as in [48]; (iv) Bicubic downsampling with scale=0.25, where the size of neighborhoods for interpolation is scale × scale as in [49]. Table 2 presents the corresponding results in terms of the *r*, LPIPS, and FID metrics. When considering these results collectively, our method demonstrates the most consistent and effective improvement across the majority of degradation types.

#### 4.5.3. Drusen Detection Results

**Figure 7:**
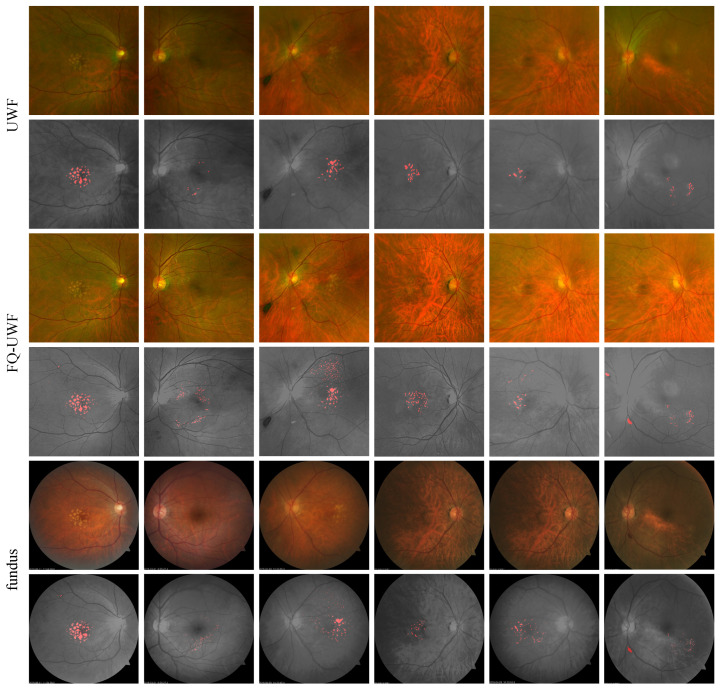
Qualitative drusen detection results.

Figure 7 presents samples of $I_{UWF}$, $I_{FQ-UWF}$, and $I_{fundus}$ images with corresponding manually annotated drusen region masks. Quantitative comparative evaluations of the drusen region masks for $I_{UWF}$ and $I_{FQ-UWF}$ are presented in Table 3. Assuming the $I_{fundus}$ drusen mask as GT, we measure the mean average precision (mAP) as the intersection over union (IoU) [50] averaged across the number of images. The increase in mAP highlights the improved diagnostic capabilities through the enhanced $I_{FQ-UWF}$ images.

### 4.6. Ablation Study

Table 1 illustrates the performance results of method variations such as the inclusion of pre-trained $G_{DE}$ through $L_{E}$ for training, the utilization of $G_{DE}$ and $G_{SR}$, and the consideration of their configuration order. When utilizing pre-trained $G_{DE}$ before super-resolution without a separate degradation enhancement process, significantly better results in terms of γ, LPIPS, and FID metrics were observed compared to cases where only super-resolution was performed. And training $G_{DE}$ via $L_{E}$ and utilizing it for super-resolution led to overwhelmingly superior results. Also, the configuration order of $G_{DE}$ and $G_{SR}$ shows a substantial numerical difference, justifying the subsequent structure of the modules.

**Figure 8:**
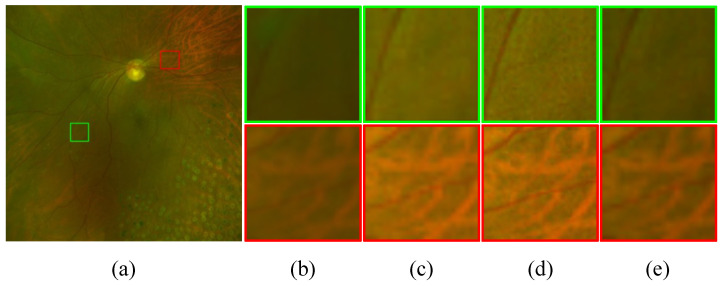
The interim improvement results (**a**) Input image, (**b**) $I_{UWF}$, (**c**) $I_{E-UWF}$, (**d**) $I_{FQ-UWF}$, and (**e**) direct super-resolution results using $G_{SR}$ of (**b**).

Table 4 shows the performance changes when specific components of the loss functions that constitute the entire network are used. According to these results, the most significant performance improvement in our model, which is composed of both $G_{DE}$ and $G_{SR}$, is achieved when fine-tuning $G_{DE}$ to suit the $I_{DS-fundus}$ image domain. Furthermore, we can observe that utilizing $G_{DE}$, even when employing the bicubic upsampling method, outperformed the results using only the SRM network. This suggests that super-resolution without adequate degradation removal has limitations in enhancing retinal images. Figure 8 illustrates the importance of the process for removing degradations before super-resolution. We can see that using the improved $I_{E-UWF}$ through the $G_{DE}$ to generate $I_{FQ-UWF}$ showcases a significantly superior enhancement capability compared to generating $I_{FQ-UWF}$ directly from $I_{UWF}$ without the prior degradation removal process.

## 5. Discussion

The proposed method can be trained on unpaired UWF and fundus image sets. By reducing dependency on paired and annotated data, our method becomes more pragmatic for integration into real-world medical settings, where the acquisition of such data is often a logistical challenge. The enhanced image quality facilitated by our approach holds the potential to significantly improve diagnostic accuracy. The ability to detect subtle changes in the retinal structure, often indicative of early-stage pathologies, is critical for timely interventions and effective disease management.

Despite the promising outcomes, our study prompts further investigation into several critical areas. The robustness and generalizability of our model need to be rigorously examined across a spectrum of imaging conditions, including instances with various ocular pathologies and diverse qualities of image acquisition. The influence of different imaging devices and settings on our model’s performance demands scrutiny to ensure broad applicability in clinical settings.

To validate the real-world impact of our enhancement method, collaboration with domain experts and comprehensive clinical validation are imperative. Ophthalmologists’ insights will provide essential perspectives on how the enhanced image quality translates into improved diagnostic accuracy and treatment planning. The feasibility of implementation in diverse clinical settings warrants further exploration considering factors such as computational requirements, integration with existing diagnostic workflows, and user-friendly interfaces for healthcare professionals.

## Figures and Tables

**Table 1:** Quantitative evaluation of KBSMC dataset.

Method	*r* ↓ (*p*-Value)	LPIPS ↓ (*p*-Value)	FID ↓ (*p*-Value)
ZSSR [13]	0.775 (<0.001)	0.624 (<0.001)	117.193 (<0.001)
*cycle-in-cycle* GAN [36]	0.803 (<0.001)	0.552 (<0.001)	103.010 (<0.001)
KMSR [37]	0.590 (<0.001)	0.435 (<0.001)	**15.192 (<0.001)**
CinCGAN [16]	0.726 (<0.001)	0.653 (<0.001)	89.511 (<0.001)
RLrestore [14] + bicubic upsampling	0.514 (<0.001)	0.595 (<0.001)	54.118 (<0.001)
Ours: $G_{DE}$ w/o $L_{E}$ → bicubic upsampling	0.520 (<0.009)	0.318 (<0.001)	30.991 (<0.001)
Ours: $G_{DE}$ w/ $L_{E}$ → bicubic upsampling	0.499 (<0.001)	0.297 (<0.001)	25.120 (<0.001)
Ours: $G_{DE}$ w/o $L_{E}$ → $G_{SR}$	0.503 (<0.001)	0.284 (<0.001)	27.055 (<0.001)
Ours: $G_{SR}$ only	0.654 (<0.001)	0.305 (<0.001)	41.317 (<0.001)
Ours: $G_{SR}$ → $G_{DE}$ w/o $L_{E}$	0.671 (<0.001)	0.300 (<0.001)	26.114 (<0.001)
Ours: $G_{SR}$ → $G_{DE}$ w/ $L_{E}$	0.585 (<0.001)	0.288 (<0.001)	26.017 (<0.001)
Ours: *full*	**0.317**	**0.231**	17.235

Values are mean ± standard deviation. For γ, LPIPS, and FID, smaller values indicate better performance. Bold values denote the most effective method corresponding to each evaluation metric.

**Table 2:** Quantitative comparison on degraded KBSMC dataset.

Degradation Type	Methods	*r* ↓	LPIPS ↓	FID ↓
Gaussian Blur (σ=7)	ZSSR [13]	0.724	0.836	137.739
	*cycle-in-cycle* GAN [36]	0.799	0.889	140.350
	KMSR [37]	0.509	0.802	49.957
	CinCGAN [16]	0.710	0.790	92.041
	RLrestore [14] + bicubic upsampling	0.663	0.811	98.818
	Ours	0.471	0.599	31.535
Illumination (γ=0.75)	ZSSR [13]	0.632	0.777	109.176
	*cycle-in-cycle* GAN [36]	0.601	0.818	104.073
	KMSR [37]	0.456	0.659	23.717
	CinCGAN [16]	0.643	0.751	79.990
	RLrestore [14] + bicubic upsampling	0.589	0.612	88.235
	Ours	0.375	0.363	20.532
JPEG Compression (rate=0.25)	ZSSR [13]	0.721	0.809	119.501
	*cycle-in-cycle* GAN [36]	0.638	0.829	90.1199
	KMSR [37]	0.557	0.771	26.181
	CinCGAN [16]	0.699	0.832	84.595
	RLrestore [14] + bicubic upsampling	0.600	0.793	91.932
	Ours	0.497	0.552	34.172
Bicubic Downsampling (scale=0.25)	ZSSR [13]	0.703	0.813	163.115
	*cycle-in-cycle* GAN [36]	0.637	0.847	112.752
	KMSR [37]	0.553	0.728	36.114
	CinCGAN [16]	0.729	0.797	104.969
	RLrestore [14] + bicubic upsampling	0.581	0.607	82.032
	Ours	0.413	0.595	39.001

Values are mean ± standard deviation. For γ, LPIPS, and FID, smaller values indicate better performance. Bold values denote the most effective method corresponding to each evaluation metric and each degradation type.

**Table 3:** Quantitative drusen detection results.

Image Pair	mAP
$I_{UWF}$-$I_{fundus}$	46.3%
$I_{FQ-UWF}$-$I_{fundus}$	62.4%

**Table 4:** Ablation study.

Loss Combination	*r* ↓	LPIPS ↓	FID ↓
$L_{H}$	0.683	0.508	81.392
$L_{H}+L_{E}$	0.415	0.329	37.508
$L_{R}+L_{E}$	0.301	0.256	23.125
$L_{R}+L_{E}+L_{M}$	0.317	0.231	17.235

Values are mean ± standard deviation. For γ, LPIPS, and FID, smaller values indicate better performance. Bold values denote the most effective method corresponding to each evaluation metric.

## Data Availability

The original contributions presented in the study are included in the article, further inquiries can be directed to the corresponding authors.

## Abbreviations

The following abbreviations are used in this manuscript:

UWF	Ultra-Widefield
FQ	Fundus Quality
GAN	Generative Adversarial Network
DE	Degradation Enhancement
SR	Super Resolution
KBSMC	Kangbuk Samsung Medical Center
IoU	Intersection over Union
GT	Ground Truth
